# The Role of Disordered Ribosomal Protein Extensions in the Early Steps of Eubacterial 50 S Ribosomal Subunit Assembly

**DOI:** 10.3390/ijms10030817

**Published:** 2009-03-02

**Authors:** Youri Timsit, Zahir Acosta, Frédéric Allemand, Claude Chiaruttini, Mathias Springer

**Affiliations:** 1 Laboratoire de Cristallographie, Institut de Biologie Physico-Chimique CNRS, 13, rue Pierre et Marie Curie, 75005 Paris, France; E-Mail: zahiracosta@gmail.com; 2 Laboratoire de Biochimie, Institut de Biologie Physico-Chimique CNRS, 13, rue Pierre et Marie Curie, 75005 Paris, France; E-Mail: frederic.allemand@ibpc.fr (F.A.); claude.chiaruttini@ibpc.fr (C.C.); Mathias.Springer@ibpc.fr (M.S.)

**Keywords:** Flexibility, structural transitions, helix-coil, calmodulin, linker, electrostatic, helix unwinding, unfolding

## Abstract

Although during the past decade research has shown the functional importance of disorder in proteins, many of the structural and dynamics properties of intrinsically unstructured proteins (IUPs) remain to be elucidated. This review is focused on the role of the extensions of the ribosomal proteins in the early steps of the assembly of the eubacterial 50 S subunit. The recent crystallographic structures of the ribosomal particles have revealed the picture of a complex assembly pathway that condenses the rRNA and the ribosomal proteins into active ribosomes. However, little is know about the molecular mechanisms of this process. It is thought that the long basic r-protein extensions that penetrate deeply into the subunit cores play a key role through disorder-order transitions and/or co-folding mechanisms. A current view is that such structural transitions may facilitate the proper rRNA folding. In this paper, the structures of the proteins L3, L4, L13, L20, L22 and L24 that have been experimentally found to be essential for the first steps of ribosome assembly have been compared. On the basis of their structural and dynamics properties, three categories of extensions have been identified. Each of them seems to play a distinct function. Among them, only the coil-helix transition that occurs in a phylogenetically conserved cluster of basic residues of the L20 extension appears to be strictly required for the large subunit assembly in eubacteria. The role of α helix-coil transitions in 23 S RNA folding is discussed in the light of the calcium binding protein calmodulin that shares many structural and dynamics properties with L20.

## 1. Introduction

In the past decade, the functional importance of intrinsically unfolded proteins “(IUP)” has been well recognized [1–6]. IUPs lack intrinsic globular structures or contain long disordered segments under physiological conditions. They participate in many functions, including cell cycle control, transcriptional and translational regulation and supramolecular assembly. Functional protein regions without a well defined 3D structure have been also found in RNA and protein chaperones [7]. The IUPs sequences are characterized by a low sequence complexity and an amino-acid compositional bias, with a low content of bulky hydrophobic amino acids and a high proportion of charged amino-acids [1–6]. Genomic analysis of disordered proteins predicts that the proportion of genome encoding IUPs increases with the complexity of the organism. More than 30% of eukaryotic proteins are either completely or partially disordered [1,2,8]. Two structurally different groups of IUPs have been identified on the basis of their physical properties: intrinsic coils and premolten globules [3]. Intrinsic coils have hydrodynamic dimensions typical of random coils in poor solvent and do not possess any ordered secondary structure. In contrast, premolten globules are more compact and display some amount of residual structure, although they are less dense than native proteins. However, as unstructured proteins generally escape detailed structural analysis, little is known about their degree of order and their conformations in the unfolded state. Such detailed information is, however, indispensable for explaining the role of disorder in biological function. A general property of IUPs is they undergo a structural transition to a folded form when they interact with their target ligands. Coupling of folding and binding or mutually induced folding events seem to be the key molecular mechanisms required for IUP functions [4,5,7,9]. It is thought that synergistic folding confers structural adaptability for the recognition of multiple binding partners. Order-disorder transitions in chaperone may also assist the correct folding of their targets in helping to avoid kinetic traps [7]. Alternatively, it has been proposed that disorder provides a simple solution to obtain large intermolecular interfaces, but with smaller proteins, genomes and cell sizes [10].

From a functional point of view, ribosomal proteins represent an interesting category of IUPs. Indeed, one of the most surprising features of ribosomal proteins found in the crystal structures of ribosome subunits is the finding that almost half of the proteins have globular bodies with long extensions that penetrate deeply into the particle’s core [11–15]. It has been proposed that these extensions that are most often disordered in solution play a key role in ribosomal assembly [16,17]. In addition, many of ribosomal proteins are involved in translational regulation in binding to operator sites located on their own messenger RNA [18]. A challenging question is therefore: how two distinct functions can be accomplished by small proteins that are most often partly or fully disordered in the absence of their binding partners?

The present review is focused on the role of structural disorder in the early steps of the large particle assembly. Biochemical, genetical and structural data have been be put together for trying to elucidate how the extensions of the ribosomal proteins (L3, L4, L13, L20 and L22) that are essential for the first steps of the eubacterial large subunit assembly [19,20], participate to the rRNA folding pathway. We show, first, that these five proteins display different types of extensions such as loops in L3, L4 and L13, β-hairpins in L22 and a long α-helix in L20. Second, the comparison of their structures in the free and bound states shows that each of them undergoes a distinct type of structural transition that seems to be associated with a different function. Finally, the α-helix-coil transition observed in L20 appears to be only type of disorder-order transition that is strictly required for the early steps of the large particle assembly.

## 2. Ribosome Assembly

The two bacterial subunits 30S and 50S assemble into a functional 70S particle that roughly consists of two-thirds RNA and one third protein [21,22]. The small subunit contains 16S RNA and 21 “S” ribosomal proteins. The large 50 S subunit is composed of two RNA, 23 S (2904 nt) and 5 S RNA (120 nt) and 33 proteins [23]. During the course of the particle assembly, a set of RNA conformational changes and protein binding leads to particles of increasing compactness. The process is highly interactive and the binding of many proteins depends on prior binding of other proteins. Primary binding proteins bind directly and independently to rRNA. Intermediate structures of increasing compactness have been identified *in vitro*. Since similar intermediates have been observed *in vivo*, it is thought that they reveal important aspects of RNA folding and protein binding during the assembly pathway. *In vivo*, the early assembly reactions already start with a small number of r-proteins shortly after the onset of rRNA synthesis. Thus, ribosomal assembly that is coupled with transcription only takes one minute. However, ribosome assembly *in vitro* takes several hours with the need of several steps of incubation at high temperature [24].

Assembly of 30 S that is simpler and faster than 50S is now well documented [21,22,25,26]. Incubation of 16 S RNA and a complete set of proteins at low temperature produces a 21 S reconstitution intermediate. It contains 16 S RNA and primary and secondary binding proteins. Heating to 42 °C induces a conformational change that results in a 26 S particle RI*. Addition of the tertiary binding proteins leads to the formation of the 30 S particle. The assembly landscape of the 30 S subunit proceeds through a global rate-limiting conformational change and traverses a landscape dotted with various local conformational transitions [25].

**Figure 1.**
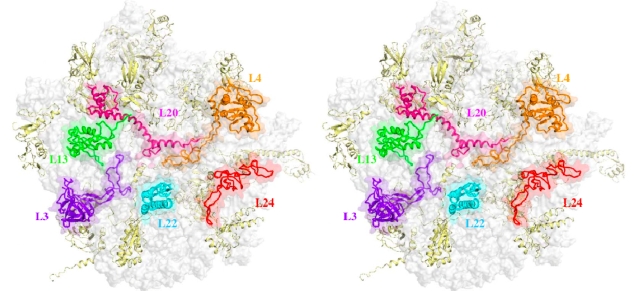
Stereo view of the large subunit of *T. thermophilus* [14] showing the distribution of the six ribosomal proteins L3 (purple blue), L4 (orange), L13 (green), L20 (magenta), L22 (cyan) and L24 (red) essential for the formation of the first reconstitution intermediate *in vitro*.

The assembly of the 50 S large particle is much more complex. An assembly map has been elaborated for the 50 S particle of *E. coli* ribosome [27,28]. Three reconstitution intermediates have been found: RI_50_(1) 33 S, RI_50_*(1) 41 S, RI_50_(2) 48 S and 50 S. Twenty two proteins are incorporated into the first intermediate RI_50_(1). During the assembly gradient, five proteins essential for the early assembly reaction (RI_50_*(1)) bind exclusively near the 5′-end of the 23S RNA. Among them, L4, L20, L22 and L24 that bind on the first rRNA domains are essential (Figure 1).

L24 that binds near the 5′-end and L3 that binds at the 3′-end of the 23S RNA, are considered as initiator proteins, since they bind independently to other r-proteins. The existence of two major protein assembly centres (L24 and L3) located at the ends of the 23S rRNA (I+II and V+VI) has been confirmed by reconstitution experiments using separate transcripts of the six major structural domains of 23S RNA [29]. This study indicates that the two centres assemble independently of each other around protein L24 and L3. Then five primary binding proteins L3, L4, L20, L22 and L24 play an essential role on the assembly of the first reconstitution intermediate. The three dimensional structures of the ribosomal particles [11–15] are in good agreement with these biochemical data. A detailed analysis of the proteins of the large subunit of *H. marismortui* has provided interesting structural insights on 23S assembly [16]. (i) Larger protein/RNA interface seems to correlate with proteins that bind early in the course of assembly. (ii) Component buried in the interior must bind the assembling ribosome earlier.

## 3. Do Ribosomal Protein Extensions Play a Role in Subunit Assembly?

Although the biological role of the extensions is still unclear, it has been postulated that they could participate to ribosome assembly on the basis of the crystal structures of the ribosome subunits [16]. The extensions of ribosomal proteins often lack obvious tertiary structure and in many regions are also devoid of significant secondary structure. While the globular domains are found on the particle’s exterior, the extensions penetrate deeply into the subunit core and are intertwined with rRNA helices (Figure 1). As a consequence, most of the proteins that contain extensions do not crystallise in the free state. When their crystallisation is possible, the extensions are generally not visible in the electron density map since they are disordered. The detailed analysis of the ribosomal proteins of the large particle of *H. marismortui* has brought many structural insights that support this hypothesis [16]. First, the extensions are basic and flexible, a property that make them candidates for assembling RNA segments during rRNA folding. In both subunits, these extensions have a distinctive amino acid composition and they differ from the globular domains mainly in glycine (13.7% vs. 8%), arginine (15,9% vs. 7.5%) and lysine (12,7% vs. 5.1%) [16,17]. The basic nature of the extensions enables them to neutralize the highly negatively charged RNA backbone. The higher glycine contain is supposed to increase their flexibility and to avoid steric clashes in tightly packed RNA regions [16]. Second, it has been noted that extensions that represent only 18% of the proteins are responsible for 44% of the total RNA surface buried by protein interaction. Because they make many contacts with rRNA and often interact with more than one domain of the RNA, it is thought that one role might be the stabilization of the proper RNA tertiary structure. Third, the finding of extensions in proteins essential (L3, L4, L22 and L20) for the formation of the first intermediate RI_50_ (1) in *in vitro* [27,28] reconstitution experiments has suggested that they may participate to ribosome assembly. Another possible role of the absence of secondary structure in the extensions is that it could allow interactions in major grooves of RNA double helices that are not wide enough to accommodate larger elements of protein secondary structures such as α-helices [16,17].

This hypothesis fits well with current views on protein/RNA interactions in which induced fit or co-folding are required for the assembly [30,31]. Many other examples of order-disorder transitions have been observed in protein/RNA interactions and growing evidence has shown that intrinsically unstructured proteins (IUPs) participate to many assembly and regulation functions [1–7]. Following this view, co-folding or disorder/order transition in r-proteins extensions would help to avoid the kinetic traps that frequently impede the correct RNA folding during the course of ribosome assembly [7]. The examination of subunit crystal structures also suggests that the globular domains of the assembly proteins bind first to rRNA [16,17]. Then, the extensions would bind additional segments in different domains, thus contributing to approach and seal distant rRNA regions. However, steric considerations require that proteins that contain extensions bind 23 S RNA at a stage prior to the formation of significant tertiary structure. Otherwise, the extensions would not have access to their binding sites. Therefore, the binding of extensions must occur before final assembly of the surrounding parts of the subunit.

## 4. A More Complex Picture: Different Categories of Extensions May Have Distinct Functions

However, a deeper analysis suggests that the picture may be more complex. Indeed, the structure of the protein extensions within the crystal of the ribosome particles provides a view of the final product of the assembly. These data are therefore insufficient to have an idea of the pathway of the folding of 23 S RNA. We believe that the comparison of the free and bound forms of ribosomal proteins may provide useful insights on the molecular events occurring during rRNA-protein binding. They can help to understand how induced fit or co-folding with their RNA target may assist the subunit assembly. Indeed, key steps of the rRNA folding process may involve structural rearrangements upon rRNA/protein binding [30,31].

Also, recent genetic, biochemical and structural data have shown that r-protein extensions are not systematically required for the subunit assembly. For example, the fact that some r-proteins that possess extensions are not essential for ribosome assembly indicates that they are not strictly correlated with an assembly function. It is also important to note that in the 30S subunit, none of the primary binding proteins has the extended basic tails. Rather, they appear to be typical globular proteins [17]. Moreover, in the 50 S particle, although the assembly initiator protein L24 is devoid of secondary and tertiary structure, it does not have an extension that penetrates in the ribosome core. L24 is bound at the ribosome surface similar to the other globular domains of other ribosomal proteins.

What about proteins essential for 50 S subunit assembly? Deletion mutants of the extensions should bring a clear answer to their function in assembly. The effect of the deletion of the extensions of L4 and L22, two primary binding proteins that are essential for the 50 S subunit assembly [19] has been tested *in vivo* [32]. Both proteins bind initially to domain I and are essential for the formation of the first reconstitution intermediate RI_50_*(1). Surprisingly, this study has shown that the extended loop of L4 and β-hairpin of L22 are not only dispensable for assembly into 50 S ribosomal particle but also for the proper assembly of proteins that bind later in 50 S assembly pathway [32]. These experiments provide a clear demonstration that the globular domains of these two proteins are sufficient to initiate the assembly of the large 50 S particles. In consequence, this finding does not support the general concept that extensions of ribosomal proteins play a role in ribosome assembly. Another study has also shown that C-terminal tails of S9 and S13 are not essential for ribosome functions [33]. However, these two proteins are not essential for the early steps of the 30 S subunit assembly.

Among the proteins that are essential for the large subunit assembly, L20 represents a particular case. L20, which is one of the most basic proteins of the eubacteria, is a primary binding protein that belongs to the five proteins essential for first reconstitution steps *in vitro* [34,35]. L20 can also replace the assembly initiator protein L24 for the initiation of assembly at low temperatures [36]. L20 has been also shown to be essential *in vivo*, as a deletion within its gene is lethal [37]. More importantly, deletion experiments have shown that the N-ter extension is strictly required for normal ribosome assembly [37]. To our knowledge, L20 is the sole example for which the extension is strictly required for the assembly of the large ribosome subunit *in vivo*. Thus, biochemical data clearly indicate that all the extensions of ribosomal proteins do not play a similar role. What does distinguish the extension of L20 from the ones of L4 and L22 that could explain its specific function in ribosome assembly?

**Figure 2.**
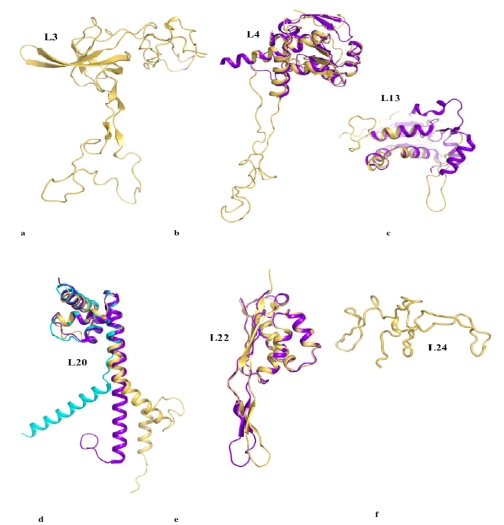
Comparison of the six proteins that are essential for the early steps of the assembly of the 50 S subunit of the eubacterial ribosome. a. L3. b. L4. c. L13. d. L20. e. L22. f. L24. The structures of the proteins bound to the ribosome are depicted in yellow and the free forms, when available, are depicted in purple blue. In the crystal structure of the free L20, two folded states coexist within the unit cell: a partially unfolded form (cyan) and a folded form (purple blue) (see also Figure 3).

Figure 2 compares the structures of the six proteins L3, L4, L13, L20, L22 and L24 that are essential for the early steps of the 50 S particle assembly. When available, the coordinates of the structures of the free forms are superimposed on the forms bound to the 23 S RNA within the the 50 S subunit of *Thermus thermophilus* [14]. Except L24 that is totally devoid of secondary structure and does not have any extension, three categories of extensions can be distinguished. The first category contains the proteins L3, L4 and L13. Their ribosome bound forms display ordered extension loops that are totally devoid of secondary structure. Within the 50 S particle, these loops are visible and well ordered due to their intermolecular interactions with the 23 S RNA. However, in the crystal structures of their unbound states (purple blue on Figure 2), the inner loops of L4 (68 aas, arg45–lys103) and of L13 are fully disordered [38,39].

**Figure 3.**
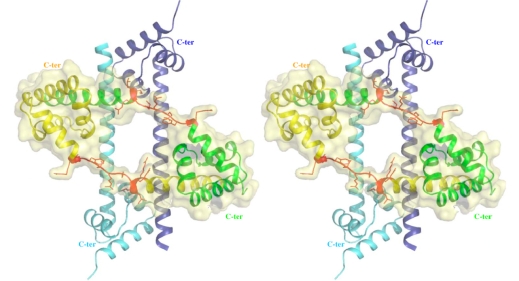
Crystal structure of unbound L20 protein. Stereoview of the heterotetramer of the asymmetric unit. The two folded monomers are represented by blue and cyan ribbons. The two partly unfolded monomers are represented by green and yellow ribbons. The unfolded regions of the α2 helix are represented in red. The folded and unfolded dimers mutually stabilise each other.

**Figure 4.**
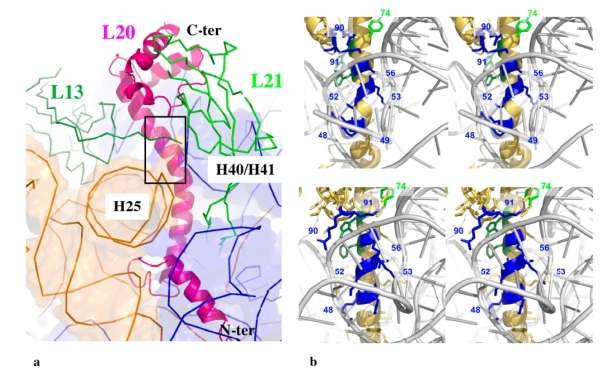
(a) The path of L20 within the core of the large subunit of *T. thermophilus* [14] (as a representative structure for eubacteria). The box represents the region that is zoomed in b. (b) The interaction of the basic residues (blue) of cluster of the helix α2 of L20 with the 23 S RNA within the 50 S subunit of *E. coli* (up) [15] and of *T. thermophilus* (bottom) [14]. The numbering of residues is shifted to fit to the sequence of *A. aeolicus* (-2 aas).

Thus, in the first category, a disordered loop becomes simply an ordered loop upon rRNA binding, without any change in the secondary structure. Although no structural data are available for the free form of L3, it is likely that its extension should be also fully disordered in the absence of RNA. In contrast, in the second category, the L22 extension (23 aas, glu78–ser101) that consists of a β-hairpin displays a similar structure in the free [40] and the bound form [14] (Figure 2e). Here, the extension that is structured without the binding target does not undergo a structural transition upon rRNA binding. In the third category represented by L20, the bound form of the N-terminal extension (60 aas) is structured into a spectacularly long α-helix α2 and a smaller N-terminal α1 helix that penetrate deeply into the ribosome core (Figure 2d, 4). The crystal structure of the unbound form of L20 has revealed the coexistence of two folding states within the unit cell (Figure 2d, Figure 3) [41]. In the folded one, the long helix α2 is fully formed and straight. In the unfolded one, α2 is unwound and stretched from arg 48 to arg 57, a conserved cluster of basic residues. Thus, in the third category, the extension undergoes a coil-helix transition in a specific region during the binding to the 23 S RNA. This analysis suggests that the specific role of L20 extension in ribosome assembly may be related to its particular structural and dynamics properties.

## 5. The Role of Coil-helix Transition of the L20 Extension in 23 S RNA Folding

Within the eubacterial 50 S subunits, L20 is bound at the interface of two RNA domains and interacts with the helix H40/41 on one side and the helix H25 on the other side (Figure 4a) [13–15]. While its globular C-ter domain interacts with L21 and L13 at the surface of the ribosome, its long α-helical extension seals the approach of two domains of the 23 S RNA. The charged side chains of the cluster of the basic residues interact with the phosphate groups of the H40/41 helix backbone (Figure 4b). It is clear that these residues participate in RNA recognition and charge neutralisation of the phosphate groups of the 23 S RNA. However, the crystal structure of the unbound form has shown that this specific region is unstructured before reaching this final bound state. Consequently, the dynamic properties of the extension may also play a critical role in the early steps of the 50 S subunit assembly. A detailed analysis of its properties is therefore important for elucidating its specific function.

**Figure 5.**
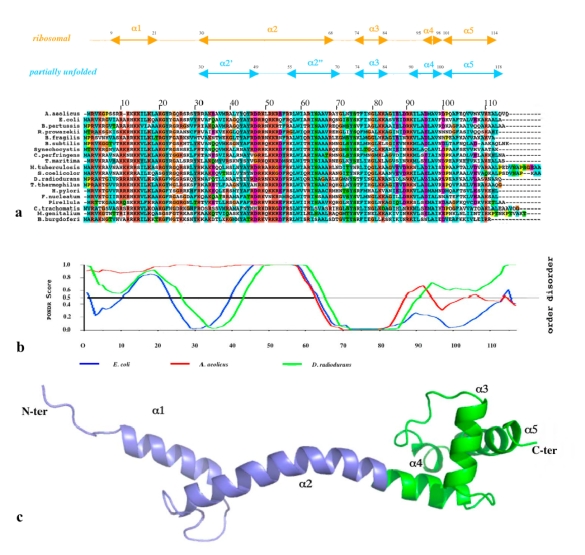
(a) Sequence alignment of the ribosomal protein L20 of representative species of eubacteria. The corresponding secondary structure elements are indicated above in cyan for the partially unfolded free crystal form and in orange for the folded ribosomal form. (b) Disorder prediction calculated with the program PondR for three species. Red: *A. aeolicus.* Blue; *E. coli.* Green: *D. radiodurans.* (c) Ribbon representation of the ribosomal form of *T. thermophilus.* The N-ter extension is represented in blue and the C-ter globular domain is represented in green.

**Figure 6.**
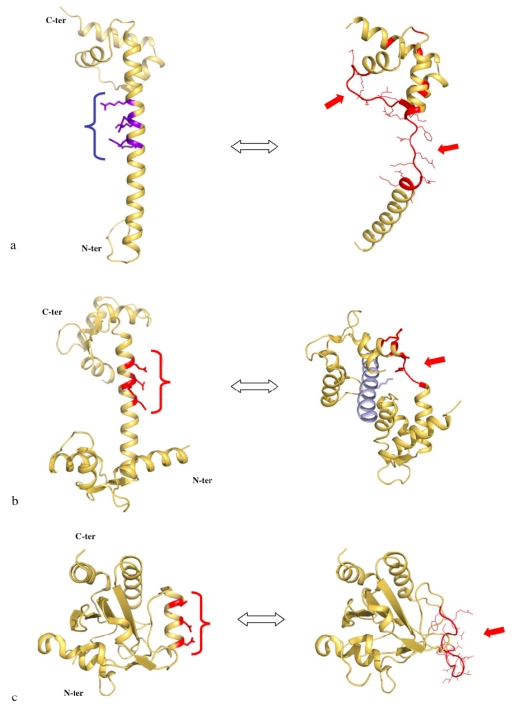
Charge repulsion and unwinding of α-helices. Comparison of the folded (left) and the partially unfolded (right) forms of L20 (2GHJ)(a), calmodulin (1CCL and 2IX7) (b) and poplar thioredoxin (2P5Q and 2P5R)(c). The cluster of charged residues are indicated by parentheses. The unfolded regions are indicated with an arrow. In each case, the electrostatic repulsion between the side chains of the charged residues is thought to be responsible for the helical unwinding. The unfolded form of L20 is stabilised through intermolecular contacts with the folded form in the crystal packing (Figure 3).

While a NMR study has proposed that the extension of L20 is totally unstructured in the absence of rRNA [42], the crystal structure of the unbound form [41] and disorder prediction programs such as PondR [43] indicate that both the extension and the C-ter globular domain are disordered in discrete regions (Figure 5 and 6a, right).

How to explain the discrepancy between NMR and crystallographic data? The absence of peak corresponding to the N-terminal extension in the NMR spectrum [42] may be rather the signature of the existence of an equilibrium between two protein conformations than an indication of its complete unfolding (J. Dyson, personal communication). It is therefore likely that the crystallisation has trapped two L20 conformations pre-existing in solution. Having them together in identical physico-chemical conditions allowed their unbiased structural comparison and provided useful insights for understanding the structural transitions between them. This comparison has first, revealed the electrostatic origin of the unwinding of α2 and second, suggested a structural communication or coupling of the folding events between the N-terminal and the globular C-terminal domains. These two properties relate L20 to the well-known calcium binding protein: the calmodulin [44,45].

### 5.1. Electrostatic Origin of the Local Unwinding

The L20 extension is unwound in a cluster of phylogenetically conserved basic residues (arg 48 – arg 57). In the corresponding region of the folded form, the side chains of the basic amino acids point on the same side of the α-helix along three helix turns (Figure 6a, left). Therefore, electrostatic repulsion between the positively charged side chains is likely responsible for the instability of this helical segment. Indeed, the spatial arrangement of the side chains along the same side of three α-helix turns generates a very high density of positive charges. A similar organisation of negatively charged residues is found in the α-helical linker of calmodulin [46] (Figure 6b, left). In calmodulin, the cluster of negative charged residues is also characterized by a high instability that confers to the protein the ability to fit to many different binding partners [44,45]. The local unwinding of the linker allows structural changes that completely reshape calmodulin that can then wrap tightly around its binding target, such as the IQ motif of mysosin V [47] (Figure 6b, right). Two recent crystal structures of poplar thioredoxin peroxidase have also illustrated the role of electrostatic repulsion of charged side chains in α-helix unwinding [48]. In this enzyme, a similar distribution of negative charges along the α2 helix occurs once a cysteine (cys 92) is present in its thiolate charged form during catalysis (Figure 6c, left). The repulsion of the side chains also leads to the complete unwinding of the helix (Figure 6c, right).

It seems therefore likely that this particular distribution of charged residues along the α-helices is responsible for their higher flexibility that may have a functional role. Interestingly, in both calmodulin and L20 protein, the charged residues have a double role: conferring a local flexibility and recognizing the binding target.

### 5.2. Structural Communication between Domains

**Figure 7.**
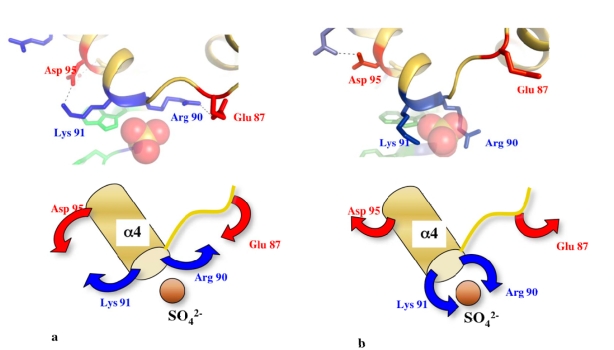
Structural rearrangements found at the interface between the globular domain and the extension of L20 of *A. aeolicus*. (a) The partially unfolded form is stabilised by the formation of two salt bridges that link two evolutionary conserved basic residues arg 90 and lys 91 to asp 95 and glu 87 located within the globular domain. (b) In the folded form, the two salt bridges are disrupted and the side chains of arg 90 and lys 91 embrace a sulfate ion. The sulfate ion occupies a position identical to a phosphate group of the H40/41 helix within the 50 S subunit of the eubacterial ribosome. In the unfolded form (a), due to the polarity of the helix (α4), the sulfate ion is also maintained at its N-ter extremity.

The comparison of the two unbound L20 forms has also revealed that not only the N-ter extension but also the C-ter globular domain display unstructured regions (Figure 6a, right). This may therefore indicate a coupling between the folding events of the two domains. A detailed analysis of the side chain conformations at the interface of the two domains indicated a possible switch mechanism allowing a structural communication between the C-terminal globular domain and the N-terminal extension (Figure 7). In other words, the folding or unfolding events in the N-terminal domain can be transmitted to the C-terminal domain.

**Figure 8.**
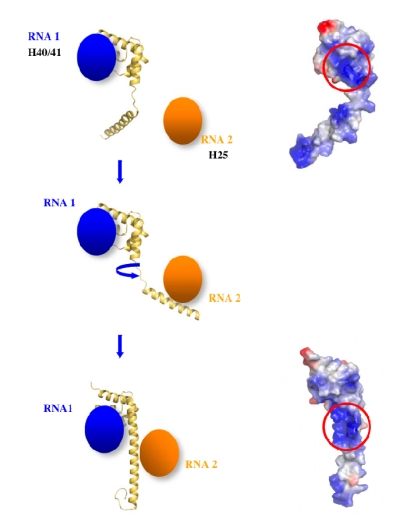
Fishing mechanism that can help to bring into proximity RNA segments coming from different rRNA domains. The two forms of L20 display very different electrostatic surface potential and affinity for RNA (top: partially unfolded, bottom: folded).

Two transient salt bridges that involve two conserved basic amino acids (arg 90 and lys 91 in N-ter of α4) at the interface of the two domains “stabilize” the partially unfolded form. Indeed, their formation seems to be related with the partial unfolding of the extension. The disruption of these salt bridges upon complete protein folding generates a dramatic reorganisation of the surface electrostatic potential (Figure 7 and 8). Thus, the two forms have different binding properties and affinities for RNA. It has been speculated that the distinct structural and dynamics features of the two folding states play distinct roles during the different steps of the folding pathway of the 23S RNA [41]. Once again, the structural coupling between the two domains of L20 is reminiscent to the properties of calmodulin. Indeed, many biophysical and mutational studies have shown that calcium binding on one globular domains triggers conformational changes and modify the stability of the central linker that in turn transmit the structural information to the other globular domain [49 and references cited therein]. Thus, long solvent exposed helices that connect two domains can be more than passive or just flexible linkers. Moreover, for the particular cases of L20 and calmodulin, the linker sequence has been finely designed by the evolution for providing a tunable flexibility that may respond to structural changes in the neighbouring domains.

### 5.3. From Dynamics to Function

Why could the coil helix transition that occurs in L20 extension essential for the early steps of 23 S RNA folding? Several hypotheses may be proposed.

(i) The specific coil-helix transition in L20 extension may help to avoid the kinetic traps during the folding of the helix H40/41 and H25. This idea is supported by a recent NMR study that shows that the rRNA target of L20 adopts a different structure in the free and bound form [50]. Thus, L20/RNA co-folding process would lower the energy required for the structural rearrangements of the RNA site required for the subsequent steps of 23 S RNA folding. This idea is supported by the finding that basic peptides whose sequence are reminiscent to L20 α2 helix can selectively trigger conformational changes in their RNA binding target [51].

(ii) Another possibility could be that the coil helix transition would be required for bringing distant rRNA segments into close proximity during the course of assembly (Figure 8). Indeed, at the beginning of the folding process rRNA is more flexible and less compact. Such a fishing mechanism has been proposed in endocytosis [52]. Indeed, the size of an unstructured peptidic segment that is longer than an α-helical one can interact with more distant partners.

(iii) The transient unfolding of the long α-helix may be required for fitting into the extremely narrow groove of the H40/41 helix junction (Figure 4b). The resulting structure would shield the electrostatic repulsion between the phosphate groups that delineate the extremely narrow groove of the L20 binding site of the H40/41 junction.

## 6. Conclusions

In this paper, the extensions of ribosomal proteins L3, L4, L13, L20 and L22 that are essential for the early steps of the large subunit assembly have been compared. This analysis sheds light on the existence of three structural categories of extensions that undergo different folding pathways. This study reveals that each category of extension has distinct roles. The coil-helix transition observed in the long basic extension of L20 that belongs to the third category could play a critical role in the 23 S RNA folding. Helix unwinding occurs in a discrete region whose flexibility is specifically tuned by a phylogenetically conserved distribution of basic residues. A similar helix-coil transition that occurs in the linker of calmodulin plays an essential role in the recognition of its multiple structurally distinct binding partners. This study relates two distinct proteins that can share similar mechanisms for accomplishing their functions.

In contrast, the inner loop of L4 that belong to the first category is not required for the early steps of the folding of the 23 S RNA. The co-folding process that leads from a disordered loop to an ordered one upon L4/rRNA association is therefore not essential for ribosome assembly. Thus this kind of transition has a less specific role than coil-helix transitions during rRNA folding. It is possible that the ordering of a disordered loop may be just required for shielding the negatively charged RNA backbone. Following this view, the deletion of this loop can be easily compensated by the binding of cations. It would be interesting to know what would be the phenotype associated with the deletion of the L3 extension. A prediction would be that the deletion of L3 extension that shares structural similarities with L4 would not affect ribosome assembly.

## Open Questions

A question that seems unsolved until today is why L20 that is an essential protein in eubacteria is not present in archea and eukaryotes? How do archea and eukaryotes compensate the absence of L20 during the early steps of the large subunit assembly? A careful structural comparison of the large subunit structure of *H. marismortui* and the ones of eubacteria would provide some structural insights. Interestingly, H25, one of RNA binding site of L20, is longer in archea. It would be then interesting to introduce the missing RNA part within eubacterial ribosome that relieve the essential character of L20.

Another question about L20 is who binds first? The extension or the globular domain? A current view is that that the globular domains of r-proteins bind first to rRNA. However, knowing that the subunit assembly is co-transcriptionally dictated by the rRNA synthesis, it is possible that in the case of L20, the extension would bind first. Indeed, H25, the binding site of L20 extension is well in 5’ relatively to the H40/41 helix junction along the rRNA transcript. H25 is therefore available before H40/41 for binding during the co-transcriptional folding of 23 S RNA.
